# Creatinine level variation in patients subjected to contrast-enhanced tomography: a meta-analysis

**DOI:** 10.1590/1677-5449.200161

**Published:** 2021-07-05

**Authors:** André Brusamolin Moro, João Gabriel Nakka Strauch, Anderson Dillmann Groto, Jeferson Freitas Toregeani

**Affiliations:** 1 Centro Universitário Fundação Assis Gurgacz – FAG, Cascavel, PR, Brasil.; 2 Clínica de Radiologia – UNITOM, Cascavel, PR, Brasil.; 3 Universidade Federal do Paraná – UFPR, Toledo, PR, Brasil.; 4 Universidade Estadual do Oeste do Paraná – UNIOESTE, Cascavel, PR, Brasil.

**Keywords:** kidney diseases, contrast media, tomography

## Abstract

Variation in the creatinine levels of patients who have undergone contrast-enhanced computed tomography (CT) has been adopted as a practical method for assessment of possible kidney damage caused by the contrast. Criteria employed include an absolute increase in serum creatinine ≥ 0.5 mg/dL or a relative increase ≥ 25% as indicative of possible renal disorders, such as contrast-induced nephropathy (CIN). Our objective was to analyze the incidence of CIN by means of a meta-analysis of nine articles related to incidence of kidney damage caused by contrast, calculating odds ratios (OR) and confidence intervals (95%CI) using RStudio. The overall incidence of CIN in patients who had CT scans was 11.29%, with an OR of 1.38 (95%CI 0.88–2.16). Non-ionic contrasts are safer than other types of contrast, and volumes exceeding 115 mL may be associated with CIN. Preexisting kidney disease had a statistically significant relationship with worse CIN rates.

## INTRODUCTION

Many diseases are often diagnosed or monitored using computed tomography (CT) scans. These examinations can require administration of iodinated contrast media to improve definition and visualization of anatomic structures, particularly the blood vessels.

Contrasts are substances employed to increase or reduce the density of an organ or cavity during radiological examinations by attenuating the X-rays and are widely used in computed tomography, magnetic resonance, and digital subtraction angiography.^1^

Contrast agents can be classified on the basis of dissociation and release of particles with electrical charges (ionic and non-ionic) and on the basis of their osmolality (Table 1): High Osmolar Contrast Media (HOCM), compounds with 4 to 7 times the osmolality of blood, Low Osmolar Contrast Media (LOCM), or Iso-Osmolar Contrast Media (IOCM).^1^^,^^2^

**Table 1:** Description of contrast media.

**Description**	**Chemical formula**	**Osmolality**	**Example**
Ionic monomer	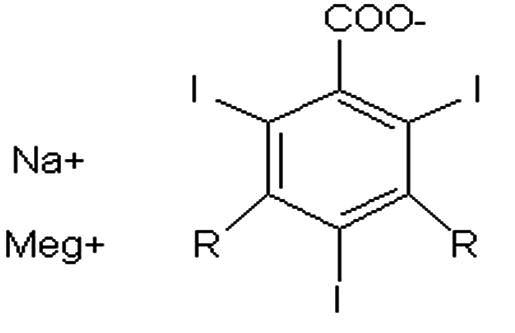	High osmolality	Metrizoate (Isopaque®)
Ionic dimer	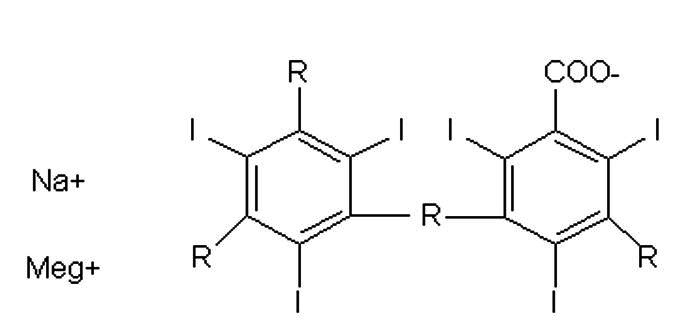	Low osmolality	Ioxaglate meglumine (Hexabrix®)
Non-ionic monomer	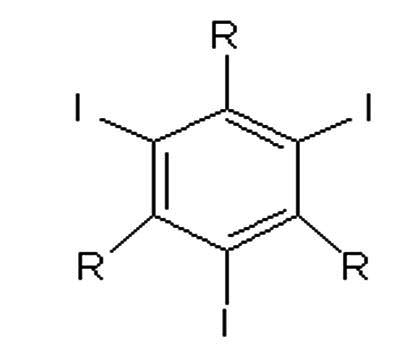	Low osmolality	Iohexol (Ominipaque ®)
			Iomeprol (Iomeron®)
			Iopromide (Ultravist®)
			Iopamidol (Isovue®)
Non-ionic dimer	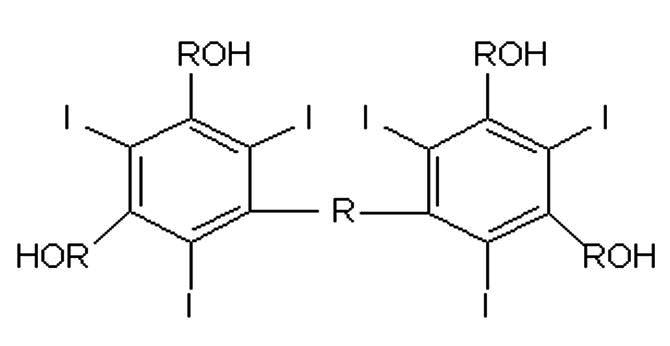	Iso-osmolality	Iodixanol (Visipaque®)

These substances are metabolized via glomerular filtration, with a mean clearance half-life in the range of 90 to 120 minutes in healthy individuals.^3^ The main adverse reactions observed are cardiovascular (anaphylactic shock), pulmonary (bronchospasm), otorhinolaryngological (laryngeal edema), and dermatological (itching and cutaneous edema).^4^

Contrast use can trigger a condition known as contrast-induced nephropathy (CIN), which is characterized by sudden deterioration of renal function and related to administration of iodinated contrast media.^5^

Contrast-induced nephropathy can be defined as an absolute increase in serum creatinine ≥ 0.5 mg/dL or a relative increase ≥ 25%.^6^^,^^7^ In addition to the factors mentioned above, correct classification of CIN must consider the temporal relationship between creatinine elevation and exposure to contrast agents and other causes of kidney damage must be ruled out.^5^

The pathophysiology of CIN consists of sudden renal dysfunction with onset 24 to 72 hours after administration of contrast media (CM).^8^^,^^9^ Although controversial, it is believed that CM are responsible for induction of renal vasoconstriction, which is the main cause of the renal ischemia and tubular toxicity.^10^^,^^11^ Finally, the ischemia causes reactive oxygen species (ROS) to form, which compound the ischemia and exacerbate kidney damage, preventing filtration and, primarily, tubular reabsorption.^5^^,^^6^

The incidence of CIN can vary between patients, depending on the presence or absence of risk factors for acute kidney damage. Among patients with risk factors, the incidence of CIN can be as high as 50%.^11^^,^^12^ The principal non-modifiable risk factors for development of renal problems after use of contrast are diabetes mellitus, advanced age, preexisting renal failure, and coexisting heart and/or liver disease.^12^ Modifiable factors include the volume of contrast agent employed, hypotension, dehydration, use of diuretics, and nonsteroidal anti-inflammatories.^8^^,^^13^^,^^14^

For CIN prophylaxis, radiology services employ volume infusion (Ringer lactate or physiological saline) before administration of contrast; infusion of N-acetylcysteine; and control of the volume of contrast employed.^5^ With regard to the prophylaxis method, it is recommended that volume infusion is given by intravenous administration of 0.9% physiological saline (PS) at 100 mL/h, from 6 to 12 hours before contrast is administered and 4 to 12 hours afterwards,^5^ while N-acetylcysteine can be given at a dosage of 1,200 mg diluted in 100 mL of 0.9% PS, administered 2 hours before contrast and 10 to 18 hours afterwards.^11^

Even when the reduction in renal function is minor, CIN is a clinical condition that can provoke a need for hemodialysis and can significantly increase morbidity and mortality, irrespective of the patient’s risk factors.^12^

The clinical relevance of the subject is founded on the lack of studies with control groups who were not exposed to contrast media.^5^^,^^13^^,^^15^ Moreover, a considerable proportion of the clinical studies that do exist recruited critically ill or hospitalized patients or people with other acute conditions that could impair renal function, making it difficult to determine relationships of causality between use of contrasts and CIN.^16^ In view of this, this article aims to employ meta-analysis to determine the true relationship between CIN and the CM employed in tomographic examinations. The conclusions, odds ratios (OR), and confidence intervals of the studies included were used to calculate the data and draw the conclusions presented in this article.

## MATERIALS AND METHODS

### Selection of studies

**Figure 1:**
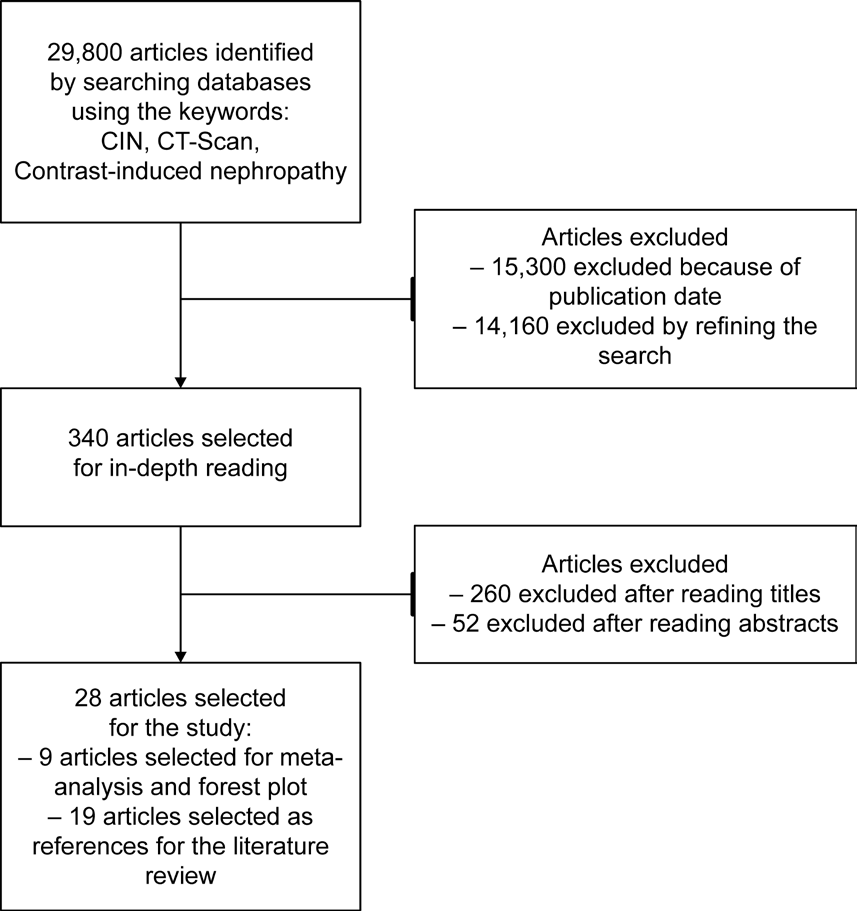
Flow diagram.

A wide-ranging search for studies was conducted on the PubMed, Scielo, and Google Scholar platforms. Searches for articles employed the keywords “CIN” (Contrasted Induced Nephropathy), “CT-Scan” (Computed Tomography scan), “contrast-induced nephropathy”, and “tomography”. A total of 29,800 articles (Figure 1) were identified. Of these, 15,300 were excluded because of publication date and 14,160 were excluded by refining the search, excluding those without the keywords and those involving the pediatric population. Of the remaining 340, 260 were excluded after reading the titles and 52 after reading the titles and abstracts.

Of the remainder, nine were selected to produce the forest plot and 19 as bibliographic references for the literature review. The remaining two references were obtained from websites with for use in the literature review.

The inclusion criteria were as follows: articles published from 2010 to 2020, with control and intervention populations, that analyzed the relationship or absence of relationship between creatinine elevation and rates of acute post-contrast kidney damage, and only investigated an adult population. Only articles published in Portuguese, Spanish, or English were included. The exclusion criteria were as follows: studies published before 2010, without control and intervention populations, or studies investigating pediatric populations. Studies that did not fit these criteria were excluded or used for the purposes of literature review.

Scientific studies analyzing prophylaxis for patients undergoing computed tomography scans were included as references. Articles that met the inclusion criteria were used in the meta-analysis, presented as a forest plot.

### Statistical analysis

The articles selected were compiled as a forest plot, calculating odds ratios and confidence intervals (95%CI) on the basis of: number of patients in the control group, number of patients in the intervention group, number of patients with CIN in the control group and number of patients with CIN in the intervention group, adopting the incidence of acute kidney damage due to contrast-enhanced tomography as the primary outcome analyzed. RStudio version 1.3.959 was used for statistical analysis and to construct the forest plot.

### Analysis and discussion of results

**Figure 2:**
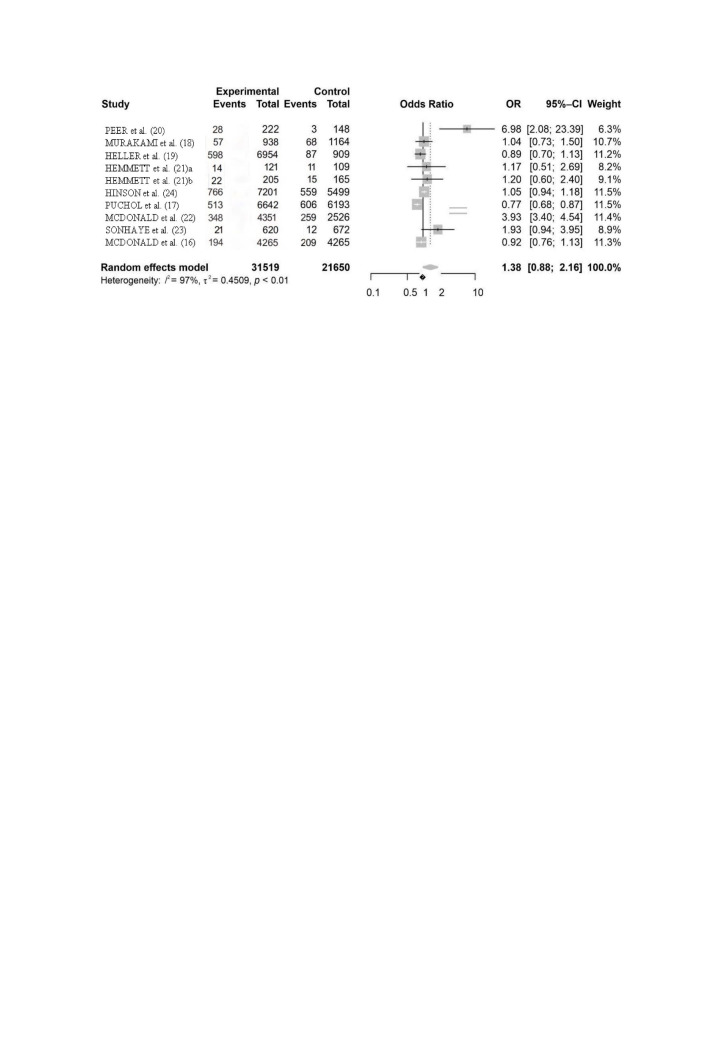
Forest plot. **OBS:** Hemmett et al.,^21^ – the first phase of the study was conducted from December 1 to December 12, 2012; Hemmett et al.,^21^ – the second phase of the study was conducted from October 1 to October 13, 2013.

Nine articles were selected for the study using the inclusion criteria.^16^^-^24 These studies analyzed a total of 53,169 patients, allocated to control and intervention groups. In all of the articles, the method used to diagnose CIN was identification of an absolute increase in creatinine ≥ 0.5 mg/dL or a relative increase ≥ 25%. Odds ratios and confidence intervals (95%CI) were calculated for all of the articles selected using the random effects model (Figure 2).

The overall incidence of CIN in this study was 11.29% for the intervention groups (administration of contrast). The overall odds ratio and 95% confidence interval in this study were 1.38 (95%CI 0.88–2.16).

With regard to the technical details of the scans conducted in the studies (Table 2), it was observed that computed tomography was used to examine a range of different anatomic areas, the most common being the abdomen, thorax, pelvis, and brain, although two studies did not specify the type of scan.

**Table 2:** Methods used to conduct contrast-enhanced tomography.

**Study**	**Type of examination**	**Contrast used**	**Volume used**	**Scientific evidence level**
Hinson et al.^24^	Unspecified contrast-enhanced CT	Non-ionic –	From 80 to 120 mL	2B
		iohexol and iodixanol		
				
McDonald et.^22^	Contrast-enhanced CT of abdomen, pelvis, and thorax	Non-ionic –	From 80 to 200 mL	2B
		iohexol and iodixanol		
				
Heller et al.^19^	Unspecified contrast-enhanced CT	Non-ionic –	100 mL	2B
		iohexol and iopamidol		
				
Murakami et al.^18^	Contrast-enhanced multi-detector CT (MDCT) of brain, neck, abdomen, pelvis and thorax	Non-ionic –	1 to 2 mL/kg up to a maximum of 150 mL, via infusion pump	2B
		iohexol, iopamidol, iopromide, iomeprol		
				
Puchol et al.^17^	Unspecified contrast-enhanced multi-detector CT (MDCT)	Unspecified low osmolar contrast	From 50 to 200 mL	2B
				
Hemmett et al.^21^	Contrast-enhanced CT of the brain, spine, abdomen, pelvis, and thorax	–	–	2B
				
McDonald et.^22^	Contrast-enhanced CT of the abdomen, pelvis, and thorax	–	–	2B
				
Peer et al.^20^	Unspecified contrast-enhanced CT	Unspecified low osmolar and iso-osmolar contrast	Mean of 115.71 mL in patients with CIN and mean of 76.15 mL in patients without CIN	2B
				
Sonhaye et al.^23^	Contrast-enhanced CT of the brain, abdomen and thorax	Non-ionic –	1.5 mL/kg up to a maximum of 150 mL	2B
		iomeprol		

* McDonald et al.^22^ and Hemmett et al.^21^ did not report the contrast used or the volume administered.

In general, the contrast type employed was non-ionic, although unspecified LOCM and IOCM were also employed.

The contrasts most used were non-ionic dimers (iodixanol) and non-ionic monomers (iohexol). The literature states that the contrasts that have the strongest association with contrast-induced nephropathy are those formulated as ionic monomer HOCM.^3^ However, the relationship between CIN and non-ionic contrasts is still uncertain,^3^ even though a study by Murakami et al.^18^ reported that non-ionic IOCM reduce the risk of post-contrast nephropathy in specific populations, such as those with diabetes mellitus and moderate renal failure. However, the study also states that this same type of contrast does not significantly reduce the risk of CIN in comparison to non-ionic LOCM.^18^

In terms of the volume of contrast used, studies report that the risk of CIN increases by 12%for each additional 100 mL administered for a coronary scan.^3^ In the present study, mean contrast media volume administered was not calculated because of a lack of uniformity among the studies in terms of the criteria adopted to describe the volume administered to patients. Comparing the results of the forest plot of incidence of CIN and the volume of contrast used, it was observed that in the study by Peer et al.^20^ a mean volume of 115 mL of contrast employed was associated with an increased risk of development of contrast-mediated kidney damage. A meta-analysis^25^ detected that the volume administered may not be so important for CIN, since the variation in the milliliters (mL) administered to different patients is low and the influence of the volume of CM on contrast-induced nephropathy is difficult to correlate.

The risk factors for CIN identified were age > 55 years, diabetes mellitus, and renal failure, reported in only one study,^23^ which is consistent with the findings of a meta-analysis by Moos et al.,^25^ apart from age, which these authors correlated with contrast-induced nephropathy in patients > 65 years of age, and use of nonsteroidal anti-inflammatories. None of the other authors listed in the forest plot reported comorbidities or age as predisposing factors for creatinine elevation.

Patients who needed hemodialysis or whose final outcome was death because of contrast were only described by Peer et al.,^20^ who reported five patients needing hemodialysis and four deaths. A 2017 study by McDonald et al.,^22^ only observed relationships with dialysis and mortality in patients with a glomerular filtration rate (GFR) ≤ 45 mL/min/1.73 m^2^. One of the reasons suggested by these authors is that patients with GFR ≤ 45 are those who have severe kidney damage and are thus more susceptible to the vasoconstrictive effects of CM. Another study, by Garfinkle et al.,^26^ reported that the risk of dialysis in patients given CM is statistically irrelevant at all levels of renal function.

A meta-analysis by Lee et al.^27^ suggests that chronic kidney disease is not a risk factor for CIN, irrespective of the patient’s GFR. It should however be noted that the article states that the lower the patient’s residual renal function, the lower the number of patients assessed and the smaller the volume of contrast employed. Moreover, few studies on the subject were analyzed, thus increasing the bias acknowledged by the authors in their conclusions.

With regard to preventative measures, Hinson et al.^24^ reported that use of prophylactic measures may be associated with the lower number of patients who met the diagnostic criteria for CIN, while Andreucci et al.^8^ reported that N-acetylcysteine reduced renal cell toxicity when ionic, low osmolar non-ionic, or iso-osmolar contrast media were administered. In contrast, Peer et al.^20^ stated that patients in their study who were given prophylactic measures before administration of CM had higher rates of CIN than those who were not, and Palli et al.^11^ demonstrated that use of prophylactics such as N-acetylcysteine and ascorbic acid were not effective for preventing CIN in critically ill patients.

McDonald et al.^16^ and other authors such as Luk et al.^13^ and Passamani et al.^28^ demonstrated that there is still a lack of studies with outpatients that include control groups, which are necessary to reduce bias and provide greater objectivity with regard to the relationship between CIN and contrasts.

Systematic reviews by Silver et al.^29^ and Corbett et al.^30^ highlight the applicability of models for predicting CIN risk. Although the first of these studies only investigated cases involving coronary angiographic procedures (rather than contrast-enhanced CT), it stated that the best performing risk prediction models of the 12 analyzed included assessment of chronic kidney disease, age, diabetes mellitus, heart failure, and hypotension or shock. The second of these reviews highlighted the favorable cost-effectiveness of implementation of a three-step testing system to classify patients requiring prophylactic measures to protect against CIN. Future studies should investigate the true applicability of these tests to contrast-induced tomography in outpatients with the objective of reducing the risk of contrast-mediated kidney injury.

The objective of the present article was to analyze studies published in the global literature to correlate the incidence of CIN with contrast-enhanced tomography. However, despite the confidence interval obtained that demonstrated the existence of such a relationship, it cannot be stated with certainty that contrast can cause this pathology because of biases in the articles analyzed.

There is therefore a need for studies that can reduce the degree of bias by: (1) conducting more studies in which patients are subdivided into control and intervention groups; (2) analyzing outpatients, excluding those who are in hospital, critically ill, or admitted via the emergency room; and (3) employing additional diagnostic criteria for kidney damage beyond elevation of creatinine levels.

This study is subject to certain limitations that should be considered. First, a large proportion of the studies that had both control and intervention groups did not apply rigorous patient selection criteria, including, in most cases, patients with other diseases that could affect the research findings, increasing bias. There is also a lack of studies with outpatients, with more controlled clinical conditions, which would reduce the influence of creatinine levels. Therefore, the results presented in this article may be overestimated.

## CONCLUSIONS

The incidence of CIN observed among patients who underwent tomography with contrast was 11.29% with OR 1.38 (95%CI 0.88–2.16).

No significant risk factors for development of CIN were identified, with the exception of preexisting kidney disease, which was related to higher risk of dialysis and death. The utility of prophylactic measures remains uncertain.

Non-ionic contrasts appear to offer greater safety with relation to development of CIN. Contrast volumes exceeding 115 mL appear to be related to increased CIN incidence. More studies are needed to improve understanding of kidney damage after administration of contrast, or its exacerbation in cases with preexisting kidney disease.
