# Supplementary material for: Creatinine level variation in patients subjected to contrast-enhanced tomography: a meta-analysis
Source: J Vasc Bras. 2021 Jul 5;20:e20200161. doi: 10.1590/1677-5449.200161 (PMC8256998; doi:10.1590/1677-5449.200161)
Supplement: Translation

# Translation: Avaliação dos níveis de creatinina em pacientes submetidos à tomografia contrastada: uma metanálise

**Published:** 2021-06-24

**Authors:** André Brusamolin Moro, João Gabriel Nakka Strauch, Anderson Dillmann Groto, Jeferson Freitas Toregeani

**Affiliations:** 1 Centro Universitário Fundação Assis Gurgacz – FAG, Cascavel, PR, Brasil.; 2 Clínica de Radiologia – UNITOM, Cascavel, PR, Brasil.; 3 Universidade Federal do Paraná – UFPR, Toledo, PR, Brasil.; 4 Universidade Estadual do Oeste do Paraná – UNIOESTE, Cascavel, PR, Brasil.

**Keywords:** nefropatias, meios de contraste, tomografia

## Abstract

A variação do valor encontrado nos exames de creatinina em pacientes submetidos à tomografia computadorizada (TC) contrastada tem sido utilizada como método prático para a avaliação de possíveis lesões renais causadas pelo uso do contraste. Entre os critérios, considera-se o aumento absoluto de creatinina sérica ≥ 0,5 mg/dL ou relativo em ≥ 25% para possíveis distúrbios renais, como a nefropatia induzida por contraste (NIC). Nosso objetivo foi analisar a incidência de NIC através de uma metanálise envolvendo nove artigos relacionados à incidência de lesão renal por contraste, sendo calculado o *odds ratio* (OR) e o intervalo de confiança (IC95%) por meio do programa RStudio. A incidência de NIC em pacientes submetidos a TC foi de 11,29%, sendo o OR de 1,38 (IC95% 0,88–2,16). Contrastes não iônicos apresentam maior segurança em seu uso que outros tipos de contraste, e o volume maior que 115 mL pode estar relacionado a NIC. A doença renal prévia apresentou significado estatístico em agravar a NIC.

## INTRODUÇÃO

Algumas doenças são frequentemente diagnosticadas ou acompanhadas por exames de tomografia computadorizada (TC). Nesses exames, é necessária a administração de meios de contraste iodados para se obter uma melhor definição e visualização das estruturas anatômicas, em especial dos vasos sanguíneos.

Os contrastes são substâncias utilizadas para aumentar ou diminuir a densidade de um órgão ou cavidade durante exames radiológicos através da atenuação recebida pelos raios X, sendo amplamente utilizados em tomografia computadorizada, ressonância magnética e angiografia com subtração digital^1^.

Os agentes contrastados podem ser classificados com base na dissociação e liberação de partículas com cargas elétricas (iônicos e não iônicos) e por meio da osmolaridade (Tabela 1): hiperosmolares (*High Osmolarity Contrast Media –* HOCM), compostos com 4 a 7 vezes a osmolaridade do sangue, hiposmolares (*Low Osmolarity Contrast Media*) e isosmolares^1^^,^^2^.

**Tabela 1:** Descrição dos meios de contraste.

**Descrição**	**Fórmula química**	**Osmolaridade**	**Exemplo**
Monômero iônico		Hiperosmolar	Metrizoato (Isopaque®)
Dímero iônico		Hiposmolar	Ioxaglato de meglumina (Hexabrix®)
Monômero não iônico		Hiposmolar	Iohexol (Ominipaque ®)
			Iomeprol (Iomeron®)
			Iopromida (Ultravist®)
			Iopamidol (Isovue®)
Dímero não iônico		Isosmolar	Iodixanol (Visipaque®)

Essas substâncias têm sua metabolização via filtração glomerular, com tempo médio de ½ vida de eliminação entre 90 e 120 minutos em indivíduos saudáveis^3^. Entre as principais reações adversas, observam-se as cardiovasculares (choque anafilático), pulmonares (broncoespasmo), otorrinolaringológicas (edema de laringe) e dermatológicas (prurido e edema cutâneo)^4^.

Nesse cenário, pode ocorrer uma condição denominada nefropatia induzida por contraste (NIC), a qual é caracterizada pela deterioração súbita da função renal, relacionada à administração de meios de contraste iodados^5^.

A NIC é caracterizada pelo aumento absoluto de creatinina sérica ≥ 0,5 mg/dL ou relativo em ≥ 25%^6^^,^^7^. Para a correta categorização da NIC, devem-se considerar, além dos fatores já mencionados, uma relação temporal entre o aumento da creatinina e a exposição a agentes de contraste e a exclusão de outras causas de lesão renal^5^.

A fisiopatologia da NIC consiste em uma disfunção súbita da função renal que ocorre entre 24 e 72 horas após a administração de meios de contraste (MC)^8^^,^^9^. Embora controverso, acredita-se que o MC seja responsável por induzir a vasoconstrição renal, a qual seria a principal causadora da isquemia renal e da toxicidade tubular^10^^,^^11^. A isquemia, por fim, causa a formação de espécies reativas de oxigênio (ROS), que, somadas à isquemia, aumentam a lesão renal, dificultando a filtração e principalmente a reabsorção tubular^5^^,^^6^.

A incidência da NIC pode variar entre pacientes, dependendo da presença ou não de fatores de risco para lesão renal aguda. Entre aqueles com fatores de risco, a incidência de nefropatia por contraste pode chegar a 50%^11^^,^^12^. Os principais fatores de risco não modificáveis para o desenvolvimento de problemas renais após o uso de contraste são o diabetes melito, a idade avançada, a insuficiência renal preexistente e doenças cardíacas e hepáticas coexistentes^12^. Entre os fatores modificáveis, encontram-se o volume de agente contrastado utilizado, a hipotensão, a desidratação, o uso de diuréticos e anti-inflamatórios não esteroidais^8^^,^^13^^,^^14^.

Como profilaxia da NIC, os serviços de radiologia utilizam a infusão de volume (Ringer lactato ou soro fisiológico) antes da administração do contraste; infusão de N-acetilcisteína e controle do volume de contraste utilizado^5^. Com relação ao método de profilaxia, sugere-se, para a infusão de volume, que ela seja realizada por meio da administração intravenosa de soro fisiológico (SF) 0,9% a 100 mL/h, de 6 a 12 horas antes da utilização do contraste e até 4 a 12 horas após^5^; já a N-acetilcisteína pode ser utilizada na dose de 1.200 mg diluídos em 100 mL de SF 0,9%, administrada 2 horas antes do contraste e até 10 a 18 horas subsequentes à utilização do MC^11^.

A NIC é uma condição clínica que pode, mesmo em baixas reduções da função renal, levar à necessidade de hemodiálise, além de, independentemente dos fatores de risco que o paciente possua, poder aumentar significativamente a morbimortalidade^12^.

A relevância clínica desse tema baseia-se na falta de estudos que envolvam grupos-controle não expostos aos meios contrastados^5^^,^^13^^,^^15^. Além disso, grande parte das pesquisas clínicas presentes envolve pacientes criticamente doentes, internados ou com outras condições agudas que poderiam agravar a função renal, dificultando uma relação de causalidade dos contrastes com a NIC^16^. Desse modo, o presente artigo visa estabelecer, por meio de uma metanálise, a real relação da NIC com os MC utilizados em exames tomográficos. As conclusões, o *odds ratio* (OR) e o intervalo de confiança dos estudos selecionados foram utilizados para a computação de dados e as conclusões do presente artigo.

## MATERIAIS E MÉTODOS

### Seleção dos estudos

Foi realizada uma ampla busca de estudos pelas plataformas PubMed, Scielo e Google Acadêmico. Foram pesquisados artigos com base nas palavras-chave “*CIN*” (*Contrasted Induced Nephropathy* – nefropatia induzida por contraste), “*CT-Scan*” (*Computed Tomography scan* – tomografia computadorizada), nefropatia induzida por contraste e tomografia. Foi encontrado um total de 29.800 artigos (Figura 1), com 15.300 deles sendo excluídos devido ao período de publicação e 14.160 por pesquisa refinada, excluindo-se artigos sem as palavras-chave e que envolvessem população pediátrica. Dos 340 restantes, 260 foram excluídos após leitura somente do título e 52 após leitura do título e do resumo.

**Figura 1:** Fluxograma.

Do restante, nove foram selecionados para a obtenção do *forest plot* e 19 para referência bibliográfica. As outras duas referências restantes foram obtidas por meio de *websites* com finalidade de referencial bibliográfico.

Os critérios de inclusão foram: artigos publicados entre os anos de 2010 e 2020, com população-controle e de intervenção, que analisassem a relação ou não do aumento da creatinina com os índices de lesão renal aguda pós-contraste e que pesquisassem apenas a população adulta. Foram incluídos apenas artigos nas línguas portuguesa, espanhola e inglesa. Os critérios de exclusão foram: estudos anteriores ao ano de 2010, sem população-controle e de intervenção ou que pesquisassem a população pediátrica. Os estudos que não se adequaram aos critérios foram excluídos ou foram utilizados para fins de referência bibliográfica.

Trabalhos científicos cujo estudo era a análise de profilaxia para pacientes submetidos à tomografia computadorizada foram incluídos como referência. Os artigos que se adequaram aos critérios de inclusão foram utilizados para o estudo da metanálise, apresentados sob forma de *forest plot*.

### Análise estatística

Os artigos selecionados foram compilados sob forma de um gráfico de *forest plot*, calculando-se o *odds ratio* e o intervalo de confiança (IC95%) por meio de: número de pacientes do grupo-controle, número de pacientes do grupo de intervenção, número de pacientes com NIC do grupo-controle e número de pacientes com NIC do grupo de intervenção, com a incidência de lesão renal aguda por tomografia contrastada sendo o desfecho principal a ser analisado. Para a análise estatística e a formulação do *forest plot*, foi utilizado o programa RStudio versão 1.3.959.

### Análise e discussão dos resultados

Dos critérios de inclusão, nove artigos foram selecionados para estudo^16^^–^ 24. No total, foram analisados 53.169 pacientes alocados em grupo-controle e grupo de intervenção. Em todos os artigos, foi constatado o método diagnóstico para NIC como o aumento absoluto de ≥ 0,5 mg/dL ou relativo de 25% dos níveis de creatinina. Foram calculados o *odds ratio* e o intervalo de confiança (IC95%), pelo modelo de efeitos randômicos, de todos os artigos selecionados (Figura 2).

**Figura 2:** *Forest plot*. **OBS:** Hemmett et al.^21^, – a primeira fase desse estudo foi realizada entre os dias 1 e 12 de dezembro de 2012; Hemmett et al.^21^, – a segunda fase desse estudo foi realizada entre os dias 1 e 13 de outubro de 2013.

A incidência de NIC nesse estudo foi de 11,29% para o grupo de intervenção (administração de contraste). O *odds ratio* e o intervalo de confiança de 95% nesse estudo foi de 1,38 (IC95% 0,88–2,16).

Com relação às especificidades técnicas dos exames utilizados nos estudos (Tabela 2), nota-se que a tomografia computadorizada foi realizada em diversas regiões, sendo as mais citadas o abdome, o tórax, a pelve e o cérebro, com dois estudos não especificando o tipo de tomografia.

**Tabela 2:** Métodos utilizados para a realização da tomografia contrastada.

**Estudo**	**Tipo de exame**	**Contraste utilizado**	**Volume utilizado**	**Nível de evidência científica**
Hinson et al.^24^	TC contrastada não especificada	Não iônico –	Entre 80 e 120 mL	2B
		iohexol e iodixanol		
				
McDonald et.^22^	TC contrastada de abdome, pelve e tórax	Não iônico –	Entre 80 e 200 mL	2B
		iohexol e iodixanol		
				
Heller et al.^19^	TC contrastada não especificada	Não iônico –	100 mL	2B
		iohexol e iopamidol		
				
Murakami et al.^18^	TC multidetectora (MDCT) Contrastada de cérebro, pescoço, abdome, pelve e tórax	Não iônico –	1 a 2 mL/kg até no máximo 150 mL por bomba de infusão	2B
		iohexol, iopamidol, iopromida, iomeprol		
				
Puchol et al.^17^	TC multidetectora (MDCT) Contrastada não especificada	Contraste hiposmolar não especificado	Entre 50 e 200 mL	2B
				
Hemmett et al.^21^	TC contrastada de cérebro, coluna, abdome, pelve e tórax	–	–	2B
				
McDonald et.^22^	TC contrastada de abdome, pelve e tórax	–	–	2B
				
Peer et al.^20^	TC contrastada não especificada	Contraste hiposmolar e isosmolar não especificado	Média de 115,71 mL nos pacientes com NIC e média de 76,15 mL nos pacientes sem NIC	2B
				
Sonhaye et al.^23^	TC contrastada de cérebro, abdome e tórax	Não iônico –	1,5 mL/kg até no máximo 150 mL	2B
		iomeprol		

* McDonald et al.^22^ e Hemmett et al.^21^ não descreveram o contraste utilizado e o volume administrado.

Em geral, o tipo de contraste utilizado foi o não iônico, sendo também utilizados MC hiposmolares e isosmolares não especificados.

Os contrastes mais utilizados foram dímeros não iônicos (iodixanol) e monômeros não iônicos (iohexol). A literatura afirma que os contrastes que possuem maior relação com a nefropatia induzida por contraste são aqueles formulados como monômeros iônicos hiperosmolares^3^. Entretanto, a relação entre NIC e contrastes não iônicos ainda é incerta^3^, apesar do estudo de Murakami et al.^18^ relatar que o uso de contrastes não iônicos isosmolares reduziram o risco de nefropatia pós-contraste em populações específicas, como aquelas com diabetes melito e insuficiência renal moderada. Além disso, o estudo cita também que esse mesmo tipo de contraste não reduziu significativamente o risco de NIC se comparado aos não iônicos hiposmolares^18^.

Quanto ao volume de contraste utilizado, estudos relatam que, a cada 100 mL a mais administrados em uma coronariografia, aumenta-se o risco de NIC em 12%^3^. No presente estudo, não foi calculado o volume médio de contraste administrado devido à falta de uniformidade entre os estudos quanto ao critério de descrição do volume aplicado nos pacientes. Comparando os resultados do *forest plot* quanto à incidência de NIC e ao volume utilizado de contraste, observa-se que, no estudo de Peer et al.^20^, a média de 115 mL de contraste utilizado obteve relação com maior risco ao desenvolvimento de lesão renal mediada por contraste. Uma metanálise^25^ identificou que o volume administrado pode não ser tão importante para a NIC, pois a variação em mililitros (mL) administrada para diferentes pacientes é baixa, sendo a influência do volume dos MC na nefropatia induzida por contraste de difícil correlação.

Os fatores de risco identificados para NIC foram idade > 55 anos, diabetes melito e insuficiência renal, relatados apenas por um estudo^23^, estando em consonância com a metanálise de Moos et al.^25^, a não ser pela idade, à qual os autores correlacionam a nefropatia induzida por contraste em pacientes > 65 anos, e o uso de anti-inflamatórios não esteroidais. Os demais autores listados no *forest plot* não relataram comorbidades ou idade como predisponentes para o aumento dos níveis de creatinina.

Pacientes que necessitaram de hemodiálise ou cujo desfecho final resultou em morte por conta dos contrastes foi relatado apenas por Peer et al.^20^, com cinco pacientes submetidos à hemodiálise e quatro que faleceram. O estudo de McDonald et al. 22, de 2017, cita a relação de diálise e mortalidade apenas para pacientes em que a taxa de filtração glomerular (TFG) é ≤ 45 mL/min/1,73 m^2^. Entre os motivos citados pelos autores, os pacientes com TFG ≤ 45 são aqueles relacionados a um comprometimento renal severo, sendo mais suscetíveis aos efeitos vasoconstritores dos MC. Outro estudo, realizado por Garfinkle et al.^26^, relata que o risco de diálise nos pacientes submetidos aos MC é estatisticamente irrelevante em todos os níveis de função renal.

A metanálise de Lee et al.^27^ sugere que a doença renal crônica não é fator de risco para NIC, independentemente da TFG do paciente. Contudo, ressalta-se que o artigo cita que, quanto menor fosse a função renal, menos pacientes eram avaliados e menor era o volume de contraste utilizado. Além disso, poucos estudos sobre o assunto foram avaliados, aumentando-se, desse modo, o viés da conclusão verificada pelos autores.

Com relação às medidas preventivas, Hinson et al.^24^ citaram, em seu estudo, que as medidas profiláticas utilizadas podem estar associadas ao número reduzido de pacientes que se enquadraram ao diagnóstico de NIC, e Andreucci et al.^8^ relataram que a N-acetilcisteína reduziu a toxicidade em células renais quando foram administrados contrastes iônico, não iônico hiposmolar e isosmolar. Por outro lado, Peer et al.^20^ afirmaram que, em seu estudo, os pacientes que receberam medidas profiláticas antes da administração de MC tiveram maiores índices de NIC do que aqueles que não receberam, e Palli et al.^11^ demonstraram que o uso de medidas profiláticas como a N-acetilcisteína e o ácido ascórbico não obtiveram êxito em prevenir os índices de NIC em pacientes criticamente doentes.

McDonald et al.^16^ e outros autores, como Luk et al.^13^ e Passamani et al.^28^, demonstram que ainda faltam estudos com pacientes não hospitalizados e que envolvam grupos-controle, necessários para a redução de viés e uma melhor objetivação da relação entre NIC e contrastes.

Revisões sistemáticas como as de Silver et al.^29^ e Corbett et al.^30^ citam a aplicabilidade de modelos preditivos de risco para NIC. O primeiro estudo, apesar de abordar apenas casos envolvendo procedimentos coronarianos (e não TC contrastada), cita que, ao analisar 12 diferentes modelos de predição de risco, os melhores destacavam-se por incluírem avaliações sobre doença renal crônica, idade, diabetes melito, insuficiência cardíaca e hipotensão ou choque; já o segundo cita o custo-benefício favorável da implementação de um sistema de diagnóstico de três fases para classificar o paciente que necessitaria de medidas profiláticas para NIC. Nesse contexto, futuros estudos poderiam abordar a aplicabilidade real desses testes em exames tomográficos contrastados nos pacientes não hospitalizados com o objetivo de reduzir os riscos de lesão renal mediada por contraste.

O presente artigo objetivou analisar estudos já publicados na literatura mundial para correlacionar a incidência da NIC com a tomografia contrastada, entretanto, mesmo com o intervalo de confiança obtido demonstrando essa relação, não pode ser afirmado com certeza que o contraste pode causar essa patologia devido aos vieses dos artigos pesquisados.

Desse modo, há a necessidade de mais estudos que possam diminuir a quantidade de vieses por meio de: (1) obtenção de mais estudos com pacientes subdivididos em grupos-controle e de intervenção; (2) pacientes analisados ambulatorialmente, excluindo aqueles internados, criticamente doentes ou admitidos em pronto-socorro; (3) utilização de mais critérios diagnósticos de lesão renal além da alteração dos níveis de creatinina.

Este estudo teve algumas limitações que devem ser consideradas. Primeiro, grande parte dos estudos que envolvem grupos-controle e de intervenção não possuem uma seleção rigorosa dos pacientes a serem estudados, envolvendo, em grande parte, pacientes com outras doenças que possam alterar os resultados das pesquisas, aumentando os vieses. Segundo, faltam pesquisas com pacientes ambulatoriais, com condições clínicas mais controladas, para diminuir a influência nos níveis de creatinina. Desse modo, os resultados obtidos pelo presente artigo podem estar sobrestimados.

## CONCLUSÃO

A incidência de NIC encontrada foi de 11,29% com OR 1,38 (IC95% 0,88–2,16) naqueles submetidos a tomografia contrastada.

Não foram identificados fatores de risco importantes para o desenvolvimento de NIC, exceto a presença de doença renal prévia, relacionada a maiores riscos de diálise e morte. A utilização de medidas profiláticas permanece incerta.

Os contrastes não iônicos aparentemente apresentaram segurança maior quanto ao desenvolvimento de NIC. Volumes de contraste maiores que 115 mL parecem estar relacionados ao aumento na incidência de NIC. Mais estudos são necessários para o melhor entendimento da lesão renal ou de sua piora em quadros com doença renal preexistente após o uso de contrastes.
